# Effect of Tai Ji and/or Qigong on patients with stable chronic obstructive pulmonary disease: A meta-analysis and systematic review

**DOI:** 10.1097/MD.0000000000041390

**Published:** 2025-01-31

**Authors:** Hongliang Liu, Ningchang Cheng

**Affiliations:** a Nanjing University of Chinese Medicine, Nanjing, China; b Department of Respiratory, Xinglong Community Health Center, Nanjing 210019, China.

**Keywords:** COPD, meta-analysis, Qigong, systematic review, Tai Ji

## Abstract

**Background:**

Chronic obstructive pulmonary disease (COPD) is a global health problem with high morbidity and mortality. Tai Ji and Qigong are traditional Chinese meditative movements, benefit COPD patient’s physical and mental health.

**Methods:**

We searched the following 7 databases Web of Science, EBSCO, Medline, PubMed, CINAHL, Cochrane Library online, and CNKI from inception to July 2023. Any RCTs managed with Tai Ji and/or Qigong on stable COPD were eligible without age, and comparison management restrict, however should be published in English. Outcome measures comprised pulmonary function, 6WMT, physical and/or cognitive function, and any assessment of people QoL.

**Results:**

Tai Ji and/or Qigong significant increased %PredFEV1 on stable COPD people (MD: 3.46, 95% CI: 1.69–5.23), and 6MWT (MD: 45.07, 95% CI: 31.16–58.97). 5/6 studies reported a meaningful change in CAT/SGRQ total (MD: −4.04, 95% CI: −7.76 to −0.32; MD: −11.95, 95% CI: −21.22 to −2.68). However, 6MWT, CAT and SGRO total were debated on high heterogeneity.

**Conclusion:**

Tai Ji and Qigong increase %PredFEV1 and promote QoL. However, the evidences are not sufficient, a proper subgroup analysis should be considered.

## 1. Introduction

Chronic obstructive pulmonary disease (COPD) is 1 sort of chronic respiratory disease characterized by progressive and partially reversible airflow limitation and presented with chronic cough and dyspnea. Acute exacerbation of COPD (AECOPD) is defined as periodic deterioration of respiratory symptoms, resulting in the need for hospitalization or urgent care, worsen patient’s lung function and quality of life even to death. COPD is a global health problem with high morbidity and mortality which led to almost 3.3 million deaths yearly and imposes a substantial economic burden that would cost worldwide economic trillions of dollars in future thirty years.^[1]^ The prevalence of COPD was 8.6% in adult, and 13.7% among individuals over forty years old in China,^[2]^ and increased gradually caused by population aged and long-time exposure under environment with high risk (tobacco, smoke or PM2.5).

The core sight on COPD is how to prevent the irreversible decrease of FVC and FEV1. Pharmacological treatment was long-termly considered as cornerstone of COPD treatment, nowadays more insights were focus on early treatment, disease stabilization and prevention of AECOPD. Patient with stable COPD was normally managed by inhaled medications presented as bronchodilators and inhaled corticosteroids (ICSs),^[3]^ however the treatment outcomes were not always satisfactory due to patient’s poor tolerance with pharmacy side effect. Pulmonary rehabilitation (PR) is described as a comprehensive intervention based on a thorough patient assessment followed by patient-tailored therapies that include exercise training, education, and behavior change, designed to improve the physical and psychological condition of people with chronic respiratory disease and to promote the long-term adherence to health-enhancing behaviors,^[4,5]^ and is an essential component of COPD management, would benefit the disease stabilization, and reduce exacerbation. Daily practice of PR included breathing training such as pursed lip breathing, yoga breathing, or breathing with computer-aided feedback, and exercise training which aimed to strengthen back, arms, and legs, as well as the muscles used to breathe.

Tai Ji and Qigong (mainly including Yijinjing, Liuzijue, Baduanjin, and others) are traditional Chinese meditative movements, originated from thousand years ago, presented as a combination with sustained mindfulness, deep diaphragmatic breathing, and gentle movements, applied as the complementary medicine in treating chronic disease, could improve patient’s cardiac and lung function, release chronic fatigue or pain, finally benefit patient’s physical and mental health. Several systematic reviews (SRs) previously revealed that Tai Ji and Qigong are owed into traditional PR, and could improve FEV1, 6WMT, and QoL for patients with COPD.^[6,7]^ The positive conclusion seemed certain, however most RCTs included in research were published in Chinese and complained as low quality due to high risk of either randomization or allocation concealment bias. Tai Ji and Qigong are becoming worldwide popularity, more studies with high quality are recently reported, therefore we tried to figure out a new protocol for study on Effect of Tai Ji and/or Qigong on patients with stable COPD.

## 2. Materials and methods

### 2.1. Study protocol

Our research was implied followed by the PROSPERO platform and Cochrane Handbook for SRs and meta-analysis, and we have registered in PROSPERO (CRD42023428833).

### 2.2. Search trails

We searched original study in the following 7 databases (details of websites were available in Supplemental Digital Content, http://links.lww.com/MD/O305): Web of Science, EBSCO, Medline, PubMed, CINAHL, Cochrane Library online, and CNKI from inception to July 2023. We searched PubMed initially used MeSH terms as “Tai Ji” AND “COPD,” or “Qigong” AND “COPD,” and then this search strategy was adopted to each database and run with familiar MeSH terms or key words.

### 2.3. Inclusion criteria

*Population:* Individuals diagnosed with stable COPD by global obstructive lung disease or other authoritative diagnostic criteria without age, publishment language and comparison management restrict.

*Interventions:* Any RCTs managed with Tai Ji and/or Qigong were eligible, and type of blind was not limited.

*Comparisons:* Any type of PR or conventional therapy or no treatment.

*Outcomes:* Outcome measures comprised pulmonary function (e.g., FEV1, FVC, FEV1/FVC%), the incidence of acute exacerbation, 6WMT, and any assessment of people QoL included the evaluation of fatigue, dyspnea, and tolerance.

### 2.4. Exclusion criteria

Participants suffering AECOPD, or case report, or conference abstract only, or study with raw data unavailable.

### 2.5. Risk-of-bias assessments

The methodological quality for the included study was assessed independently by 2 researchers (Liu, Cheng) based on Cochrane risk-of-bias criteria,^[8]^ and each quality item was graded as low risk, high risk, or unclear risk based on the following criteria: trials were considered low quality if either randomization or allocation concealment was assessed as a high risk of bias, regardless of the risk of other items; trials were considered high quality when both randomization and allocation concealment were assessed as a low risk of bias, and all other items were assessed as low or unclear risk of bias in a trial; trials were considered moderate quality if they did not meet criteria for high or low risk. The items used to evaluate bias in each trial included the randomization sequence generation, allocation concealment, blinding of participants and personnel, blinding of outcome assessment, incomplete outcome data, selective reporting, and other bias. Meanwhile we defined other bias as the different diagnostic criteria on stable COPD in each RCT.

### 2.6. Data extraction

Two lead researchers (Liu, Cheng) independently screened each study after duplicate remove and extracted the following information: name of first author, publication year, country or region of origin, participant characteristics, performance details, frequency and duration of intervention, and outcome targeted. Disagreements were resolved by consensus.

### 2.7. Statistical analysis

The association of Tai Ji and/or Qigong with stable COPD was assessed, and Tai Ji and/or Qigong was separately compared with conventional therapy or no treatment group. We performed meta-analysis to calculate risk ratios or absolute risk differences, and 95% CIs by the Mantel–Haenszel statistical method using Revman version 5.4 (Cochrane collaboration). A random-effects model was used to pool the data, and statistical heterogeneity between summary data was evaluated using the *I*^2^ statistic. Sensitivity analysis was performed when *I*^2^ > 50%, aimed to exclude low-quality studies, or trials with characteristics different from the others. We specified subgroups based on the exercise frequency of Tai Ji and/or Qigong (≥ 4 or <4 times a week), and home-based or community-based which program was taken^[9]^ (home-based, community-based, or hospital-based). All tests were 2-tailed, and *P* < .05 was considered statistically significant.

## 3. Results

**Figure 1.**
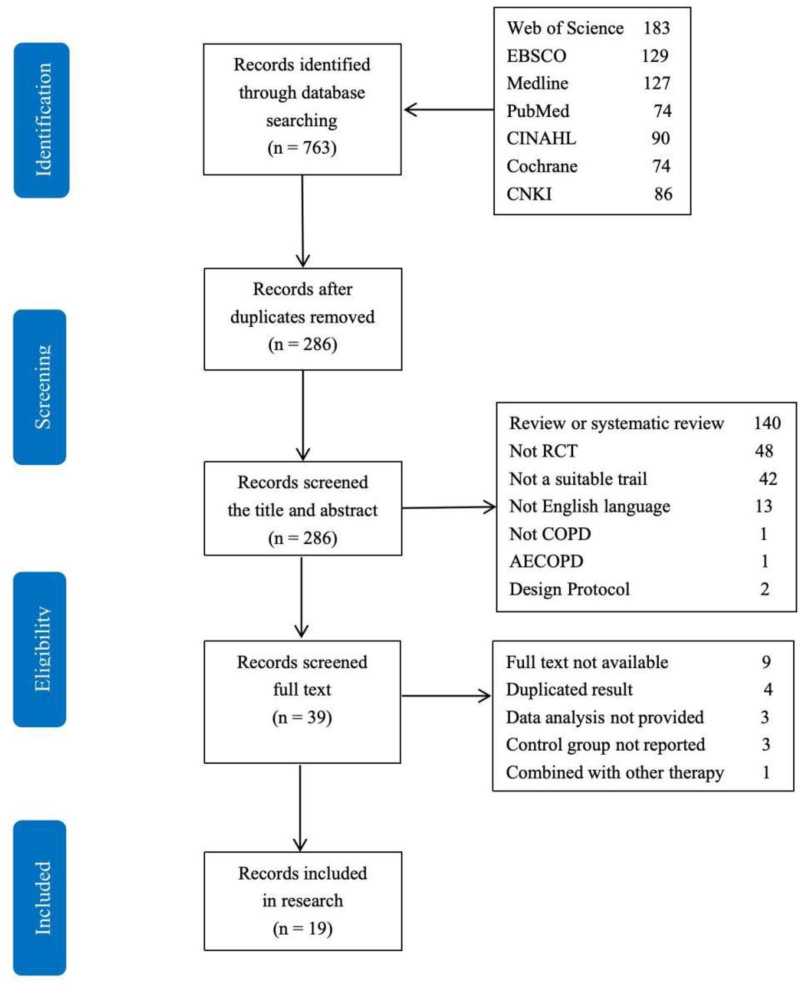
Prisma flow diagram.

In total 763 relevant trails searched from 7 databases were potentially identified, and following 477 trails were removed due to duplication. Nineteen trails (N = 1432 participants) met the eligibility criteria after screening full text (Fig. 1).

### 3.1. Study characteristics

Summary of 19 studies were presented in Table 1,^[7,10,12–28]^ including details of intervention/control group, and outcome measurement. All studies were RCT design, including 5 pilot RCTs (Dong 2021, Niu 2014, Wang 2019, Gloria 2010, Zhang 2016). Qigong/Tai Ji was separately presented as a single intervention in 7/12 studies. Eight studies adopted a complementary therapy or mixed methods as the control group, and the counterpart in other eleven studies was normal care/treatment.

**Table 1:** Characteristics of included studies.

First author	Published yr	Country	Intervention	Control*	Sample size (I/C)	Participants age mean (SD) yr (I/C)	Outcome measurements
Chan^[10]^	2011	Hong Kong, China	Tai Ji (60 min/session, 2/wk, 3 mo)	G1: Breathing and self-paced walking, G2: blank	70/G1: 69, G2: 67	71.7 (8.2)/G1 73.6 (7.5), G2 73.6 (7.4)	FVC, FEV1, 6MWT, Borg scale score
Chen^[11]^	2015	China	Breathing and Daoyin exercise (2/d, 5 d/wk, 3 mo)	Blank	30/30	57.6 (7.6)/54.2 (6.5)	%PredFEV1, FEV1/FVC, PEF%, MMEF%, mMRC, 6MWT, CAT, ESQ-COPD, total effective rate, total/major score of clinical symptoms
Dong^[12]^	2021	China	Tai Ji (30 min/session, 2/wk, 12 wk)	Cycle ergometer exercise	10/10	65.50 (6.26)/63.60 (7.88)	6MWT, SGRQ, CAT
Jiang^[13]^	2023	China	Qigong (60 min/session, 2/d, 7/wk, 3 mo) and usual care	Blank	19/18	66.11 (9.08)/64.58 (9.06)	FVC, FEV1, FEV1/FVC, %PredFEV1, %PredFVC, 6MWT, elbow isokinetic strength test, knee isokinetic strength test, SGRQ, mMRC
Kantatong^[14]^	2020	Thailand	Tai Ji (3/wk, 24 wk)	Blank	25/25	69.68 (7.67)/67.48 (10.17)	6MWT, FEV1, FVC, mMRC, SGRQ
Kraemer^[15]^	2021	America	Tai Ji (1 h/class, 2/wk, 24 wk)	Mind-body breathing*	61/31	68.6 (9.2)/67.5 (7.7)	6MWT, CRQ, 30s SST
Li^[16]^	2018	China	Liuzijue (1 h/session, 6/wk, 6 mo)	Blank	17/19	66 (9)/66 (9)	%PredFEV1, FEV1/FVC, 6MWT, SGRQ
Liu^[17]^	2023	China	Tai Ji (3/wk, 2 mo)	G1: blank. G2: total body recumbent stepper (TBRS), G3: TBRS & Tai Ji	26/G1: 26, G2: 25, G3: 25	66.27 (6.58)/G1: 60.77 (7.48), G2: 64.82 (6.04), G3: 63.04 (9.34)	FEV1, FVC, FEV1/FVC, SGRQ, 6MWT, Borg scale score, CAT, mMRC, HADS, BBS
Liu^[18]^	2021	China	Water-based Liuzijue (2/wk, 12 weeks)	G1: blank, G2: land-based Liuzijue	16/G1: 17, G2: 17	65 (11)/G1: 66 (8), G2: 65 (8)	Peak VO_2_, relative peak VO_2,_ peak min ventilatory, peak working rate, anaerobic threshold, 6MWT, 30s SST
Luo^[7]^	2023	China	Tai Ji (30 min/session, 5/wk, 52 wk)	Blank	116/110	68.03 (6.58)/67.43 (7.34)	FEV1, FVC, FEV1/FVC, exacerbation rate, SGRQ, SAS, SDS
Ng^[19]^	2011	Hong Kong, China	Baduanjin (45 min/session, 6 mo)	Breathing training	34/37	71.75 (1.05)/73.12 (1.33)	6MWT, SF-36, CRQ
Niu^[20]^	2014	China	Tai Ji (3/wk, 6 mo)	Blank	20/19	59.7 (2.76)/61.3 (2.89)	%PredFEV1, FEV1, 6MWT, PaO_2_, PaCO_2_, diaphragm strength parameters
Polkey^[21]^	2018	China	Tai Ji (1 h/class, 5/wk, 12 wk)	Standard UK practice	55/55	Data not available	FEV1, FVC, SGRQ, 6MWT, mMRC
Wang^[22]^	2019	China	Tai Ji (60 min/class, 3/wk, 3 mo)	Blank	26/24	67.83 (5.32)/67.86 (5.98)	FEV1, %PredFEV1, FEV1/FVC, 6MWT, CAT
Xiao^[23]^	2015	China	Liuzijue (45 min/session, 4/wk, 6 mo)	Blank	63/63	72.2 (1.7)/70.9 (1.4)	6MWT, CRQ, SF-36
Yeh^[24]^	2010	America	Tai Ji (1 h/class, 2/wk, 12 wk)	Blank	5/5	65 (6)/66 (6)	CRQ, 6WMT
Yeh^[25]^	2020	America	Tai Ji (1 h/class, 2/wk, 12 wk)	Blank	61/31	68.6 (9.2)/68.1 (6.7)	CRQ, 6WMT, PROMIS
Zhang^[26]^	2016	China	Yijinjing (60 min/session, 6 mo)	G1: self-management exercise, G2: blank	42/G1: 43, G2: 45	64.77 (11.07)/G1: 63.34 (7.86), G2: 62.35 (9.27)	FEV1, FEV1/FVC, %PredFEV1, 6MWT, CAT
Zhu^[27]^	2018	China	Tai Ji (5/wk, 9 mo)	Blank	30/30	67.87 (5.22)/68.10 (6.57)	FEV1, 6MWT, mMRC, CAT

G1: Group 1. G2: Group 2.

Abbreviations: %PredFEV1 = percentage predicted forced expiratory volume in 1 s, %PredFVC = percentage predicted forced volume capacity, 30s SST = 30-second chair stand test, 6MWT = 6-minute walking test, BBS = Berg balance scale, CAT = the COPD assessment test, CRQ = chronic respiratory disease questionnaire, ESQ-COPD = effectiveness satisfaction questionnaire for COPD, FEV1 = forced expiratory volume in 1 s, FEV1/FVC = the ratio of FEV1 to FVC, FVC = forced volume capacity, HADS = hospital anxiety and depression scale, mMRC = modified British medical research council, PEF = Peak expiratory flow variability, PROMIS = patient-reported outcome measurement information system, SF-36 = medical outcomes 36-item short form health survey, SGRQ = St, George respiratory questionnaire.

* Control group was time-matched with the intervention group.

### 3.2. Effect of lung function

Total ten studies reported changes in lung function post intervention, including FVC, FEV1, FEV1/FVC and %PredFEV1. Only 1 outcome, %PredFEV1, was significant different (MD: 3.46, 95% CI: 1.69–5.23, *P* < .01), which was reported by 6 studies (Chen 2015, Jiang 2023, Li 2018, Niu 2014, Wang 2019, Zhang 2016).

### 3.3. Quality of life

Multiply measurements were applied on evaluating QoL of patients with stable COPD. 6WMT was certainly considered as the primary presentation, followed with CAT, mMRC, SGRQ and CRQ. Data analysis from fifteen studies revealed that 6WMT was significant different (MD: 45.07, 95% CI: 31.16–58.97, *P* < .01), however the positive result was unavailable due to high heterogeneity (*I*^2^ = 85%). After that, a subgroup analysis was performed, and divided according to the type of intervention (Tai Ji or Qigong), but the heterogeneity remained not low. 5/6 studies reported a meaningful change in CAT/SGRQ total (MD: −4.04, 95% CI: −7.76 to −0.32, *P* < .05; MD: −11.95, 95% CI: −21.22 to −2.68, *P* < .05), however both results were debated on their high heterogeneity.

### 3.4. Certainty of evidence and reporting biases

**Figure 2.**
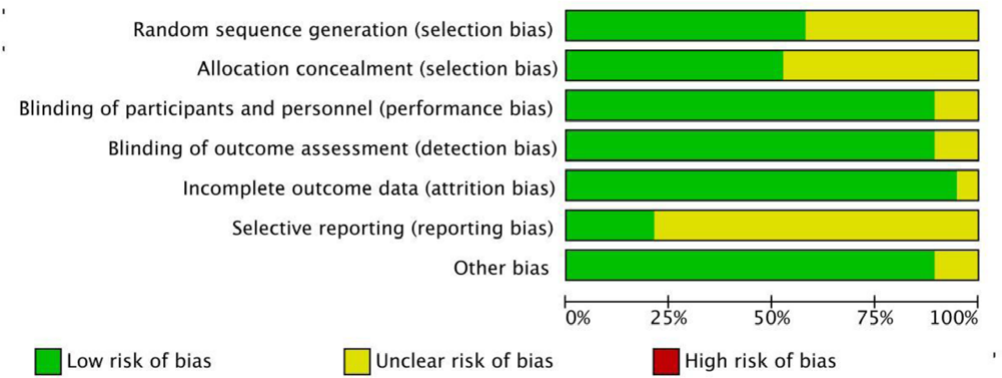
Risk of bias graph.

**Figure 3.**
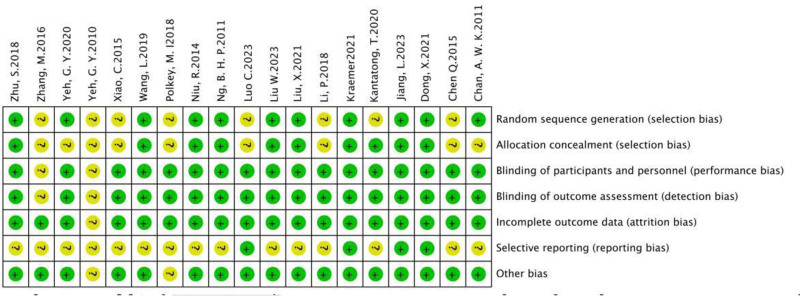
Risk of bias summary.

The risk of biases was seen in Figures 2 and 3, low or unclear risk of bias was shown in each aspect. The quality of study was variable, with 9 studies (Dong 2021, Jiang 2023, Kraemer 2021, Liu X. 2021, Liu W. 2023, Ng. B. H. 2011, Niu 2014, Wang 2019, Zhu 2018) indicating high quality, ten studies indicating moderate quality.

## 4. Discussion

The benefits to COPD patients from PR were certainly considered, included the improvement of dyspnea, health status, and exercise tolerance across all grades of COPD severity.^[5]^ Traditional PR (Tai Ji and Qigong) remained the first option in China even the world, which attributed to lower economic burden and more effectiveness than any other type of PR. Tai Ji and Qigong are mind controlled physical exercises, benefit both physical and psychological health status. A SR which included 23 studies tentatively supported that Tai chi effectively reduced anxiety and depression, and improved general mental health compared to non-mindful exercise.^[29]^ Another SR identified seventeen randomized trails implied Tai Ji and Qigong promoted cognitive function in the elderly directly/indirectly through enhancing physical function.^[30]^

Whether traditional PR is the most equivalent part is still under debate. Multiply SRs and Meta-analysis previously indicated positive result of Tai Ji and/or Qigong on physical and psychological health of COPD patient, however the results were blamed on low quality of included trails. We observed that most low-quality trails were published in non-English language. So, we put a language limit during searching trails, even CNKI, a database originated from China, only reports written in English were included. The studies included in our research were high or moderate quality, based on non-high risk on randomization or allocation concealment. However, our research did not provide strong evidence to the certain conclusion, mostly due to the high heterogeneity present in the progress of analysis. We conducted a subgroup analysis of 6MWT, the most favorable measurement of QoL, according to different intervention, Tai Ji or Qigong, the heterogeneity remained not low. We considered the duration or frequency of intervention was similar, therefore a subgroup analysis was not probable to input.

Our research updates the evidence summaries, provide a quantitative and standardized assessment of the effect of Tai Ji and/or Qigong on patients with stable COPD. Tai Ji and Qigong, primary component of traditional PR, are benefit to stable COPD people, significant increase %PredFEV1. However, the evidence of Tai Ji and Qigong on promoting QoL are not sufficient, a more proper subgroup analysis should be considered. In conclusion, Tai Ji and Qigong are the complementary management on stable COPD and presented with effectiveness and safety.

## Author contributions

**Conceptualization:** Hongliang Liu, Ningchang Cheng.

**Data curation:** Hongliang Liu, Ningchang Cheng.

**Formal analysis:** Hongliang Liu, Ningchang Cheng.

**Investigation:** Ningchang Cheng.

**Methodology:** Ningchang Cheng.

**Project administration:** Ningchang Cheng.

## Supplementary Material


