# Supplementary material for: Effect of Tai Ji and/or Qigong on patients with stable chronic obstructive pulmonary disease: A meta-analysis and systematic review
Source: Medicine (Baltimore). 2025 Jan 31;104(5):e41390. doi: 10.1097/MD.0000000000041390 (PMC11789891; doi:10.1097/MD.0000000000041390)
Supplement: Supplementary file 1 [file medi-104-e41390-s001.docx]

Supplement:

- Web of Science (<https://www.webofscience.com/>)
- EBSCO (<https://www.ebsco.com>)
- Medline (<https://www.medline.com/>)
- PubMed (<https://pubmed.ncbi.nlm.nih.gov/>)
- CINAHL (<https://www.ebsco.com/products/research-databases/cinahl-database>)
- Cochrane Library online (<https://www.cochranelibrary.com/>)
- CNKI (<https://www.cnki.net/>)

**Fig. 1. Prisma flow diagram**

**Fig. 2. Risk of bias graph**

**Fig. 3. Risk of bias summary**
